# Risk factors for secondary thyroid cancer in patients with breast cancer: a propensity‑matched SEER analysis

**DOI:** 10.1038/s41598-024-59209-x

**Published:** 2024-06-03

**Authors:** Yizhuo Diao, Ruiqi Wang, Jiaxue Cui, Chenxin Jin, Yongxing Chen, Xiaofeng Li

**Affiliations:** Department of Epidemiology and Health Statistics, Dalian Medical University, 9 Lvshun South Road, Dalian, 116044 Liaoning China

**Keywords:** SEER, Breast cancer, Thyroid cancer, COX, PSM, Cancer epidemiology, Cancer models

## Abstract

With the rapid development of imaging technology and comprehensive treatment in modern medicine, the early diagnosis rate of breast cancer is constantly improving, and the prognosis is also improving; As breast cancer patients survive longer, the risk of developing second primary cancers increases. Since both breast and thyroid are Hormone receptor sensitive organs, which are regulated by hypothalamus pituitary target gland endocrine axis, changes in body endocrine status may lead to the occurrence of these two diseases in succession or simultaneously. This study extracted clinical data and survival outcomes of breast cancer patients registered in the Surveillance, Epidemiology and End Results (SEER) database between 2010 and 2019. After matching the case and controls with propensity scores, the selected patients were randomly split into training and test datasets at a ratio of 7:3. Univariate and multivariate COX proportional regression analysis is used to determine independent risk factors for secondary thyroid cancer and construct a column chart prediction model. Age, ethnicity, whether radiotherapy, tumor primary location, N stage, M stage were identified by Cox regression as independent factors affecting secondary thyroid cancer in patients with breast cancer patients, and a risk factor nomogram was established to predict patients’ 3 and 5 year survival probabilities. The AUC values for 3 and 5 years in the training set were 0.713, 0.707, and the c-index was 0.693 (95% CI 0.67144, 0.71456), and the AUC values for 3 and 5 years in the validation set were 0.681, 0.681, and the c-index was 0.673 (95% CI 0.64164, 0.70436), respectively.

## Introduction

Breast cancer is a malignant tumor that occurs in the Epithelium of the breast gland^1^. It is the “No. 1 public enemy” that threatens the physical and mental health of women around the world today^2^. It ranks first in the incidence and death of cancer among women in most countries around the world^3^. According to the data of the 2023 American Cancer Statistical Report, breast cancer is still one of the three most common cancers among women^4^ ,occupying the first place in expected new cases among women; With the rapid development of imaging technology and comprehensive treatment in modern medicine^5^, the early diagnosis rate of breast cancer continues to improve, and the prognosis also improves^6^.The disease-free survival rate and overall survival rate of breast cancer patients have significantly improved; With the prolongation of the survival period of breast cancer patients, the risk of second primary cancer (SPC) may increase^7^; Thyroid cancer is a malignant tumor originating from thyroid follicular epithelium^8^. It is considered to be an inert tumor^9^ and the most common malignant tumor in the Endocrine system and head and neck tumors^10^; Since both breast and thyroid are Hormone receptor sensitive organs, which are regulated by hypothalamus pituitary target gland endocrine axis, changes in body endocrine status may lead to the occurrence of these two diseases in succession or simultaneously.

## Materials and methods

### Data sources

The SEER database containing 13 registry centers prepared by the National Cancer Institute was selected for the data of this study, and a total of 692,555 patients diagnosed with breast cancer from 2010 to 2019 were extracted from the database using the SEER*Stat software program, and a total of 393,722 patients were screened according to the inclusion and exclusion criteria.

### Inclusion and exclusion criteria

#### Inclusion criteria

(1) Patients diagnosed with breast cancer as the first tumor from 2010 to 2019 (2) Patients with no more than 2 tumors and the second type of tumor is thyroid cancer (3) Female patients (4) Patients with complete clinical data(Includes time of diagnosis, age at diagnosis, marital status at diagnosis, site of origin, mode of diagnostic confirmation, mode of case report, chemotherapy or not, radiotherapy or not, molecular typing, Progesterone Receptor, Estrogen Receptor, T,N, and M typing, type of secondary second tumor, interval between secondary second tumors, survival time, survival status)

#### Exclusion criteria

(1) Those with benign tumors (2) Proven only at autopsy or death (3) Patients with thyroid cancer occurring within 3 months of breast cancer diagnosis

### Independent variables

The study data were converted to categorical variables in order to make the study more intuitive and standardized. Race was categorized as black, white, and other; marital status was categorized as married, unmarried; radiation and chemotherapy status was categorized as yes, no; primary tumor location was categorized as mid-breast, quadrant I, quadrant II, quadrant III, quadrant IV, and other; estrogen receptor (ER), and progesterone receptor (PR) status was categorized as estrogen receptor (ER) and progesterone receptor (PR) status positive and negative; and molecular staging included LuminalA, LuminalB, HER2 overexpression and triple-negative; T staging as T0T1, T2, T3 and T4; N staging as N0, N1, N2 and N3; M staging as M0 and M1; and secondary thyroid cancer status as yes or no.

### Statistical methods

R4.2.3 statistical software was used for analysis. Patient standardized incidence rates (SIR) were calculated using the Multiple Principal Standardized Incidence Rates (MP-SIR) module of SEER Stat 8.4.1 software. Due to the large difference in sample sizes between the case and control groups in this study, in order to equalize the distribution of covariates between the groups, equalize confounders, and reduce selection bias, we introduced the propensity score matching (PSM) method^11^, in which the two groups of patients were matched in a ratio of 1:4^12^. Using the R software package *MatchIt*, the PSM method was used to match the case and control groups according to the year of diagnosis in a 1:4 ratio, and a total of 392,803 patients with unilateral breast cancer were included as controls, and a 1:4 matching of the case and control groups was accomplished based on the year of diagnosis of breast cancer in the 919 case group of thyroid cancer secondary to breast cancer. Patients served as controls. To ensure that the 919 cases were matched to the 3676 controls, caliper distances were chosen to be as small as possible. Propensity scores were calculated using a logistic regression model, and for better matching, the final matching caliper distance was 0.1. The standardized mean difference (SMD) was used to assess the balance of baseline information between the case and control groups after PSM. We considered SMD less than or equal to 0.1 to indicate a good match^11^.

Further univariate and multivariate analyses were performed using the COX proportional risk model to determine the risk factors for secondary thyroid cancer in breast cancer patients. R-studio software was used to randomly divide all the data into training and validation sets in the ratio of 7:3, and χ^2^ test and t-test were performed on different variables in the training and validation sets, and then univariate and multivariate Cox regression analyses were carried out on the data in the training set in order to train the model, and the validation set was used to validate the model^13^. A nomogram was created using the R packages *rms*, *foreign* and *survival* for the final filtered variables and the ROC curves were used to create the nomograms under the ROC curves. The area under the ROC curve (AUC value) and C-index were used to evaluate the accuracy of the model, the AUC and C-index range from 0 to 1, the closer to 1 indicates that the model is more accurate, and it is usually considered that the model has a better predictive ability when the AUC reaches more than 0.7; the calibration curve was used to evaluate the degree of calibration of the model, and the closer the calibration curve is to the standard curve, the stronger the predictive ability of the model. Variables with a univariate COX regression of P < 0.1 were included in the multifactor analysis, and the multifactor analysis was included in the final model with the criterion that the difference was considered statistically significant at P < 0.05^14^.

### Ethical approval and consent to participate

Not applicable. Data is available in a public database, ethics approval is not applicable.

## Results

### Standardized incidence rate

The SEER Stat 8.4.1 analysis yielded a SIR result of 14.89 with a 95% CI of 14.02–15.79 for the period 2010–2019, a predicted number of people of 74.16, and a 10 year actual number of people of 1104, with an incidence rate of 159.41/100,000, which leads to the conclusion that patients who already have breast cancer are more likely to develop thyroid cancer than healthy people.

### Propensity score matching

919 patients with thyroid cancer secondary to breast cancer after screening as case group and 392,803 patients with solitary breast cancer as control group were included in the propensity score matching, according to the year of diagnosis according to the PSM method to achieve 1:4 matching, and finally a total of 919 cases in the case group and 3676 cases in the control group were obtained, and the results are shown in Table 1. The R language *tableone* package automatically uses analysis of variance for continuous variables, which is equivalent to t-test since this data is only divided into two groups. The result before matching was P < 0.001 and the result after matching was P = 1, SMD < 0.001, which can be considered as well balanced between the matched case group and the control group.

**Table 1:** Results of PSM.

	Breast cancer then develop thyroid cacer (n = 919)	Breast cancer only, before PSM (n = 392,803)			Breast cancer only, after PSM (n = 3676)		
	Mean (SD)	Mean (SD)	p	SMD	Mean (SD)	p	SMD
Year of diagnosis	2013.96 (2.65)	2015.11 (3.03)	< 0.001	0.404	2013.96 (2.65)	1	< 0.001

### Baseline information

The 919 patients with thyroid cancer secondary to breast cancer and the 3676 patients only with breast cancer obtained after propensity score matching, for a total of 4595 patients, were included in subsequent model analyses. The baseline data of the 4595 patients are shown in Table 2.

**Table 2:** General information on the subject of the study.

	Breast cancer only, n = 3676		Breast cancer then develop thyroid cancer, n = 919	
	n	%	n	%
Age (years), mean (SD)	60.51 (12.97)		56.82 (11.45)	
Year of diagnosis, mean (SD)	2013.96 (2.65)		2013.96 (2.65)	
Race				
Black	940	25.57	64	6.96
White	2680	72.91	704	76.61
Other	56	1.52	151	16.43
Marital status at diagnosis				
Married	2066	56.20	603	65.61
Unmarried	1016	27.64	316	34.39
Radiation				
Yes	2055	55.90	558	60.72
No	1621	44.10	361	39.28
Chemotherapy				
Yes	1755	47.74	495	53.86
No	1921	52.26	424	46.14
ER				
Positive	2899	78.86	769	83.68
Negtive	777	21.14	150	16.32
PR				
Positive	1755	47.74	689	74.97
Negtive	1921	56.26	230	25.03
Breast subtype				
Luminal A	2495	67.87	651	70.84
Luminal B	457	12.43	130	14.15
HER2	195	5.30	54	5.88
TNBC	529	14.39	84	9.14
Primary site				
Central	238	6.47	47	5.11
Upper-inner	452	12.30	99	10.77
Lower-inner	196	5.33	44	4.79
Upper-outer	1388	37.76	346	37.65
Lower-outer	237	6.45	70	7.62
Other	1165	31.69	313	34.06
T				
T01	2107	57.32	518	56.37
T2	1153	31.37	310	33.73
T3	239	6.50	61	6.64
T4	177	4.82	30	3.26
N				
N0	2348	63.87	537	58.43
N1	929	25.27	275	29.92
N2	240	6.53	58	6.31
N3	159	4.33	49	5.33
M				
M0	3521	95.78	877	95.43
M1	155	4.22	42	4.57

### Comparison of baseline characteristics

The 4594 patients obtained after propensity score matching were randomly divided into training set and validation set according to the ratio of 7:3, 3219 patients in the training set and 1376 patients in the validation set. t-test was used for continuous variables, and chi-square test was used for categorical variables to characterize the intergroup differences between the training set and the validation set, and the p-values were all greater than 0.05, which proved that there were no intergroup differences in the results of the randomized splitting, and the results are shown in Table 3.

**Table 3:** Comparison of training and validation sets.

	Training set, n = 3219		Validation set, n = 1376			p
	n	%	n	%	X2 (t)	
Age (years), mean (SD)	59.91 (12.73)		59.44 (12.85)		1.124	0.2612
Race					1.726	0.422
Black	687	21.34	317	23.04		
White	2384	74.06	1000	72.67		
Other	148	4.60	29	2.11		
Marital status at diagnosis					0.007	0.9351
Married	1871	58.12	798	57.99		
Unmarried	1348	41.88	578	42.01		
Radiation					0.001	0.9729
Yes	1830	56.85	783	56.90		
No	1389	43.15	593	43.10		
Chemotherapy					0.006	0.9371
Yes	1575	48.93	675	49.06		
No	1644	51.07	701	50.94		
ER					0.202	0.6534
Positive	2564	79.65	1104	80.23		
Negtive	655	20.35	272	19.77		
PR					0.009	0.9253
Positive	2239	69.56	959	69.69		
Negtive	980	30.44	417	30.31		
Breast subtype					0.365	0.9475
Luminal A	2199	68.31	947	68.82		
Luminal B	409	12.71	178	12.94		
HER2	177	5.50	72	5.23		
TNBC	434	13.48	179	13.01		
Primary site					3.850	0.5713
Central	210	6.52	75	5.45		
Upper-inner	1198	37.22	536	38.95		
Lower-inner	220	6.83	87	6.32		
Upper-outer	394	12.24	157	11.41		
Lower-outer	164	5.09	76	5.52		
Other	1033	32.09	445	32.34		
T					0.432	0.9336
T01	1833	56.94	792	57.56		
T2	1034	112.51	429	31.18		
T3	208	22.63	92	6.69		
T4	144	15.67	63	4.58		
N					4.935	0.1766
N0	1997	62.04	888	64.53		
N1	851	26.44	353	25.65		
N2	224	6.96	74	5.38		
N3	147	4.57	61	4.43		
M					2.051	0.1521
M0	3090	95.99	1308	95.06		
M1	129	4.01	68	4.94		

### Results of univariate and multivariate cox regressions

The training set patient data were included in univariate Cox regression analysis for each of the 12 variables. To avoid omission of important variables, 11 variables with P < 0.1 in the univariate Cox regression were included in the multivariate Cox regression. We included the variables with p < 0.1 in the univariate Cox regression analysis to the multivariate Cox model to examine the independent risk factors for second thyroid cancer; when P < 0.05 in multivariate Cox regression analysis, the factor was an independent risk factor affecting patients’ secondary thyroid cancer. The results of this univariate Cox regression showed that age, ethnicity, marital status, primary tumor location, molecular typing, PR status, ER status, whether radiotherapy, whether chemotherapy, T stage, N stage, M stage were the factors affecting the secondary thyroid cancer in breast cancer patients; while the results of multivariate Cox regression showed that age, race, whether radiotherapy, primary tumor location, N-stage, and M-stage were independent risk factors affecting the development of thyroid cancer in patients with breast cancer, and the results are shown in Table 4.

**Table 4:** Results of the COX regression.

Variables	Univariate analysis			Multifactorial analysis		
	HR	95% CI	P	HR	95% CI	P
Age	0.979	0.9729–0.985	0.000	0.980	0.9731–0.9867	0.000
Race						
Black	1.000	–	–	1.000	–	–
White	3.455	2.521–4.735	0.000	3.676	2.6659–5.0688	0.000
Other	16.423	11.479–23.495	0.000	15.261	10.5857–21.9997	0.000
Marital status at diagnosis						
Married	1.000	–	–	1.000	–	–
Unmarried	0.749	0.6364~0.8813	0.000	0.955	0.8091–1.1280	0.590
Radiation						
Yes	1.000	–	–	1.000	–	–
No	0.853	0.7277~1	0.050	0.837	0.7090–0.9883	0.036
Chemotherapy						
Yes	1.000	–	–	1.000	–	–
No	0.807	0.6905–0.9423	0.007	1.0737	0.8763–1.3155	0.493
Primary site						
Central	1.000	–	–	1.000	–	–
Upper–inner	1.170	0.8078–1.694	0.406	1.353	0.9271–1.9741	0.117
Lower–inner	1.471	0.9423–2.297	0.089	1.780	1.1287–2.8061	0.013
Upper–outer	1.229	0.8133–1.858	0.327	1.517	0.9954–2.3119	0.053
Lower–outer	1.283	0.7889–2.086	0.315	1.515	0.924–2.4822	0.099
Other	1.420	0.9805–2.056	0.064	1.615	1.1096–2.3514	0.012
PR						
Positive	1.000	–	–	–	–	–
Negtive	0.775	0.6478–0.9272	0.005	0.811	0.6152–1.0687	0.137
ER						
Positive	1.000	–	–	–	–	–
Negtive	0.855	0.6964–1.05	0.136	–	–	–
Breast subtype						
Luminal A	1.000	–	–	1.000	–	–
Luminal B	1.086	0.8672–1.361	0.471	0.923	0.7272–1.1715	0.510
HER2	0.995	0.7114–1.391	0.976	1.0924	0.7150–1.6690	0.683
TNBC	0.790	0.6075–1.026	0.077	1.166	0.8075–1.6845	0.412
T						
T01	1.000	–	–	1.000	–	–
T2	1.218	1.0302–1.441	0.021	1.089	0.9076–1.3066	0.359
T3	1.136	0.8162–1.582	0.449	0.963	0.6757–1.3733	0.836
T4	1.228	0.8179–1.842	0.322	1.118	0.7159–1.7460	0.624
N						
N0	1.000	–	–	1.000	–	–
N1	1.435	1.2062–1.706	0.000	1.278	1.0517–1.5534	0.014
N2	1.170	0.8553–1.599	0.327	1.036	0.7430–1.4456	0.833
N3	1.704	1.2093–2.402	0.002	1.453	0.9907–2.1311	0.056
M						
M0	1.000	–	–	1.000	–	–
M1	2.128	1.497–3.025	0.000	1.767	1.2013–2.5979	0.004

### Creation of nomogram

**Figure 1:**
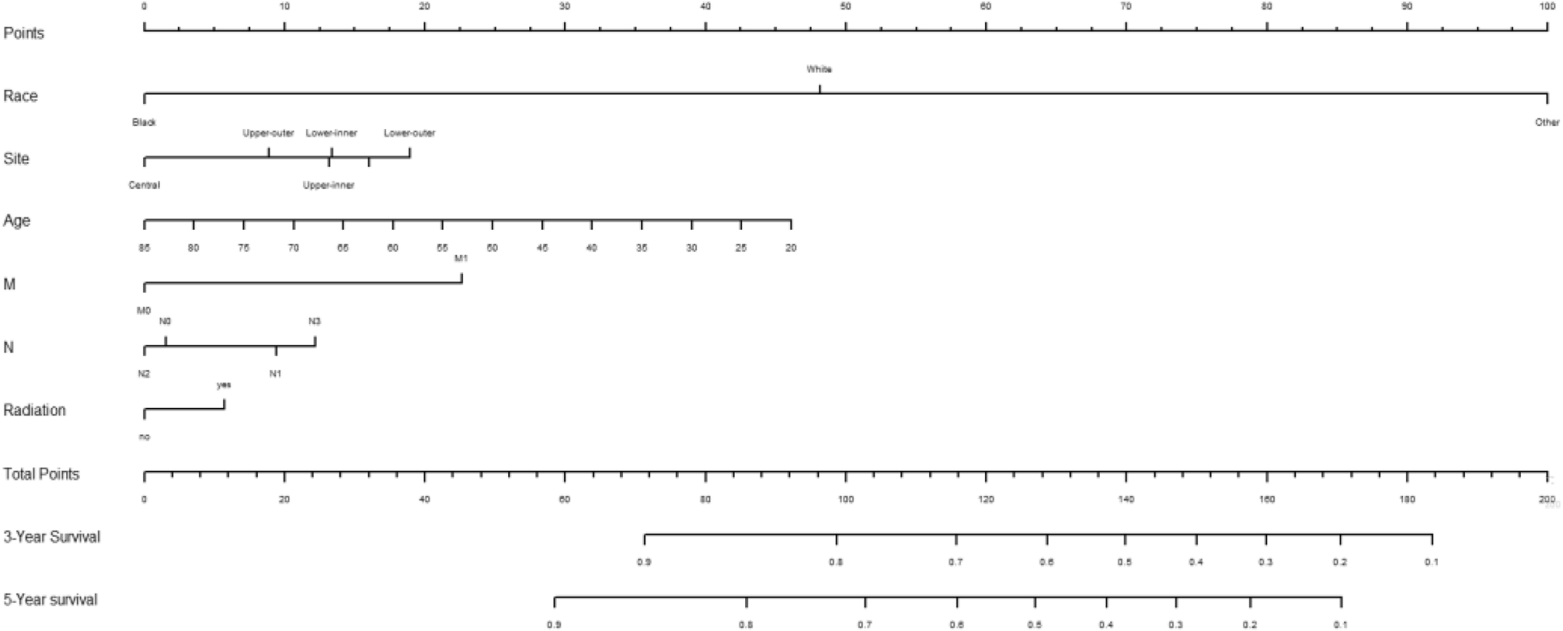
The nomogram of the COX proportional risk model.

Variables screened in the multifactorial Cox regression analysis (P < 0.05) were included in the cox proportional risk model, and nomograms were created using R-studio software. Each variable was projected upward for breast cancer patients, and then each score on the scale was summed to obtain a total score, based on which the risk of secondary thyroid cancer in breast cancer patients at 3 and 5 years could be predicted, and the higher the total score the higher the risk, the prediction results are shown in Fig. 1. Patients with breast cancer who were younger, received radiotherapy, had N1 staging, M1 staging, were white and other race, and had a primary in the inner lower quadrant were at greater risk of secondary thyroid cancer.

### Validation of nomogram

**Figure 2:**
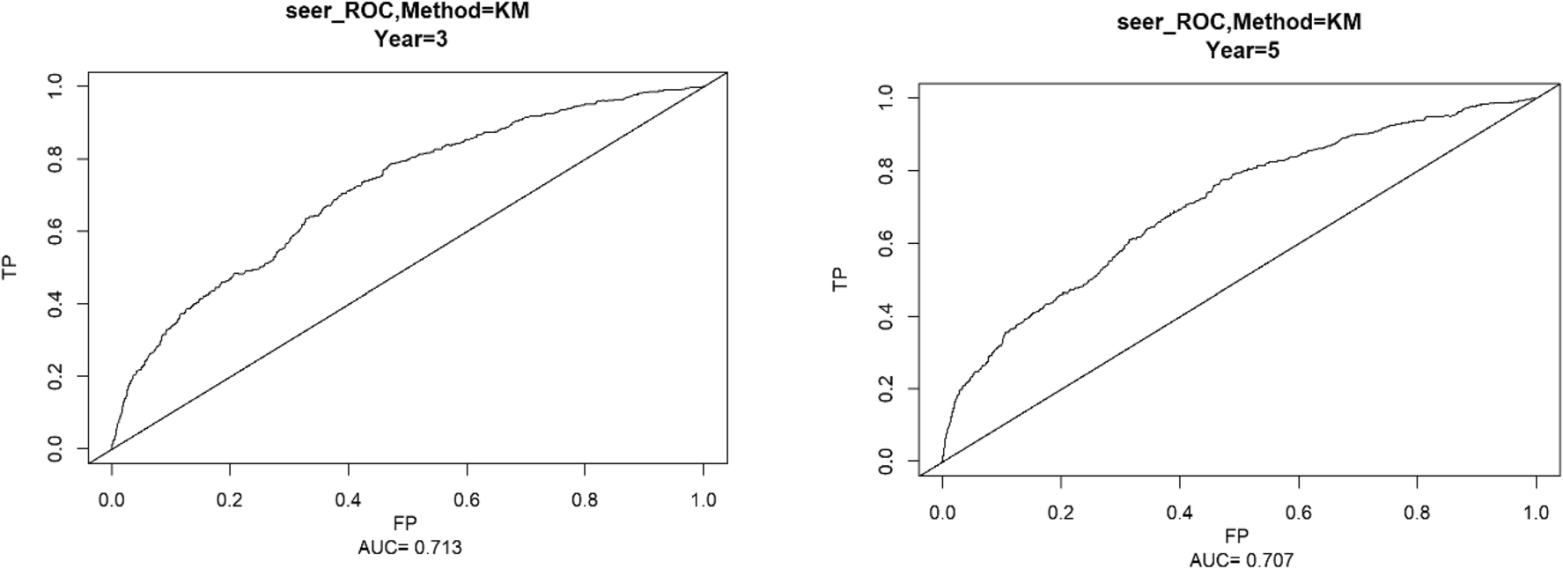
Three and Five year ROC curves for patients in the training center.

**Figure 3:**
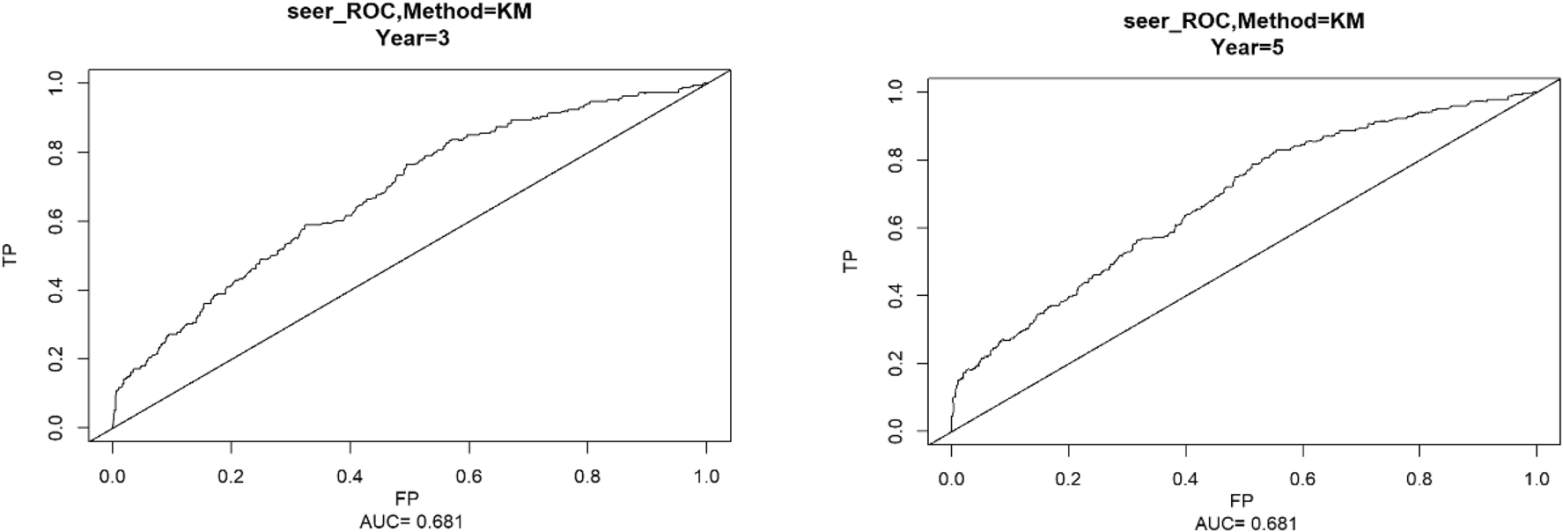
Three and Five year ROC curves for patients in the validation set.

**Figure 4:**
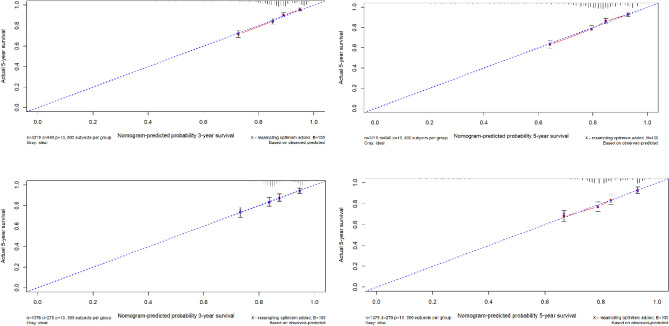
The calibration curves of 3 and 5 years.

Using the ROC curve AUC value and c-index to evaluate the differentiation of the model, we found that, in the Cox proportional risk regression model, the AUC values of three and five years for the patients in the training set (Fig. 2) were 0.713, 0.707, and the c -index was 0.693 (95% CI 0.67144, 0.71456), respectively, and the AUC of three and five years for the patients in the validation set (Fig. 3) values were 0.681, 0.681,c-index was 0.673 (95% CI 0.64164, 0.70436 respectively), we can consider the model as having moderate predictive power. The calibration plots were plotted using the data from the validation set to evaluate the fit of the established model, and the results showed that in the Cox proportional risk regression model, the calibration curves of 3 and 5 years were close to the dotted line with a 45 ° inclination in the middle, which indicated that the model fit was better.The calibration curves of 3 and 5 years are shown in Fig. 4.

### Utility and discussion

Breast cancer is known as the world’s number one “red face killer”, according to the latest cancer data released by the World Health Organization, the number of new cases of breast cancer (2.26 million) has exceeded that of lung cancer (2.2 million), and has become the most prevalent cancer in the world^4^. In recent years, more and more general hospitals have set up thyroid breast surgery departments, mainly because it is generally recognized that both the thyroid and the breast are target organs for hormone regulation by the hypothalamic-pituitary endocrine axis, and there are some common pathogenic factors in the two. Therefore, the use of a combination of clinical diagnostics and intelligent means to determine the risk factors for secondary thyroid cancer in breast cancer patients is important for both doctors and patients, and it can guide breast cancer patients for follow-up screening and help clinicians develop treatment plans for the general population and breast cancer survivors.

Although the number of breast cancer patients has been increasing year by year, the low incidence of dual primary cancers, the fact that patients with second primary cancer may be seen in different hospitals, the difficulty of data collection and the long time required for follow-up make the collection of data for the study of dual primary cancers of the breast still difficult. Therefore, this study chooses the U.S. National Cancer Database (SEER), which covers about 30% of the U.S. population, updates patient data in March to April every year, and has a large number of patients with a long follow-up period, making it a more ideal source of data for the study of dual primary cancers.

A total of 692,555 patients diagnosed with breast cancer in the SEER database from 2010 to 2019 were included in this study, and the SEERSTATA software was used to calculate the SIR > 1 for thyroid cancer secondary to breast cancer, and it can be assumed that patients with breast cancer are more likely to develop thyroid cancer compared to cancer-free populations, which is consistent with the findings of previous literature. Due to the large difference in sample size between the case and control groups, in order to minimize bias, we matched 919 patients with thyroid cancer secondary to breast cancer as the case group and 393,722 patients with solitary breast cancer as the control group using propensity score matching. Because the diagnostic criteria, examination instruments, and staging criteria may differ between years, we performed 1:4 propensity score matching according to the year of diagnosis to minimize confounding bias and to include as many control cases as possible to ensure representativeness^12^. The matched 4595 patients were randomly divided into training set and validation set in the ratio of 7:3, and the obtained training set was used to train the cox regression model, and the validation set was used to verify the stability of the model^15,16^. The results of univariate cox regression showed that the 11 factors included except ER were independent factors for secondary thyroid cancer in breast cancer patients, and the results of multivariate cox regression showed that age, ethnicity, location of primary tumor, whether or not to have radiotherapy, N-stage, and M-stage were independent factors for secondary thyroid cancer in breast cancer patients.

Contrary to the general impression that age, as a continuous variable with an OR value of less than 1, is considered a protective factor against secondary thyroid cancer from breast cancer, the younger the age, the more likely it is to be secondary thyroid cancer, and previous studies by other scholars have also demonstrated that patients with secondary thyroid cancer after breast cancer were younger compared with those who had breast cancer only^17^, which we believe may be related to the recent years of rejuvenation of the incidence of breast cancer, the early diagnosis and early treatment that makes the breast cancer patient. We believe that this may be related to the recent rejuvenation of breast cancer incidence, prolonged survival due to early diagnosis and early treatment, and the modern lifestyle, where a good prognosis increases the likelihood of a secondary second tumor. However, in some scholars’ studies, age is a risk factor for survival in patients with thyroid cancer secondary to breast cancer relative to patients with solitary breast cancer, and older patients are more likely to have lower survival rates^18^. In conclusion, age is an extremely important factor affecting breast cancer patients with secondary thyroid cancer and warrants further study.

In terms of race, the SEER database has more white profiles due to region, and multivariate cox results show that whiteness and other ethnicities are independent risk factors for secondary thyroid cancers in patients with breast cancer. This may be related to the level of medical care, conditions, and other factors that were co-incorporated into the study, with genetic susceptibility playing a different role in different factors. Mariotto A.B. et al. suggest that the higher incidence of second primary cancers among white women is due to the higher overall survival and screening rates among white women compared to black female populations, and that more comprehensive medical coverage and higher levels of medical care make it easier to diagnose the disease, which would inevitably lead to an increase in the number of diagnoses if these cancers were diagnosed at an earlier stage, which would be consistent with the results of the present study^19^. If these cancers were diagnosed at an early stage, this would inevitably lead to an increase in the number of diagnoses. However the study by Shuting Li et al. suggests that black women with breast cancer should be given attention^13^. However, in a study by Karan Seegobin et al., it was found that there was no significant difference in the incidence of secondary breast and gynecologic cancers between Caucasians and Blacks^20^ ,which may be due to the different target second primary cancer disease types in the study, and further research is needed in the future.

Breast cancer patients who receive radiation therapy are more likely to develop secondary thyroid cancers compared to those who do not, a finding that is generally consistent with current clinical opinion that radiation therapy can affect thyroid hormone secretion and thyroid function^21^. The relationship between radiation therapy and the risk of second primary cancers has long been recognized, and it has been demonstrated that radiation therapy is associated with an increased risk of second primary malignancies after exposure^21^. In several observational studies of breast cancer follow-up, the incidence of subsequent secondary acute myeloid leukemia was increased in patients with breast cancer, which may be related to the dose intensity of chemotherapy, the use of adjuvant radiotherapy, and the use of granulocyte colony-stimulating factor (GCSF)^22^. Data from the DBCG (The Danish Breast Cancer Cooperative Group) registry estimate that the proportion of second primary cancers after breast cancer associated with radiation therapy is about 9%. This is consistent with the results from the US SEER database registry^23^. However Grantzau’s analysis found that breast cancer radiotherapy was associated with a small but significant increase in the risk of second cancers for lung, esophageal, and soft tissue cancers, but was not significantly associated with second cancers for thyroid cancer^24^. Another study on the health of the population in Taiwan compared the risk of TC in BC patients who received radiotherapy and those who did not, and the risk of TC in women who received radiotherapy was not significantly higher than that in women who did not receive radiotherapy. This may be related to the selection of data from different ethnic groups in different regions.

In addition, some researchers have suggested that the ER/PR signaling pathway may be a common etiology of breast and thyroid carcinogenesis, and studies of its mechanisms, including the ER pathway and CHEK2 gene mutations in thyroid tissues. In our study, only PR receptor status was an independent factor for secondary thyroid cancer in breast cancer patients. Some researchers have suggested that patients with secondary thyroid cancer have a higher rate of both ER and PR positivity than patients with breast cancer only^25,26^.ER has two isoforms, Erα and Erβ, and overexpression of ERα in thyroid cancer tissues and lack of expression of ERβ in peripheral tissues was reported in 2011^27^ , and under-expression or deletion of ER can be considered a hallmark of thyroid cancer^28^. Undifferentiated thyroid stem and progenitor cells express lower levels of ERβ compared to differentiated human thyroid cells^29^. Therefore, low levels of ER expression may suggest dedifferentiation of thyroid cancer^27,30^. The fact that this paper did not conclude that ER has an effect on secondary thyroid cancer in breast cancer patients may be related to the fact that gene-related variables were not included in this study, and that the SEER database contains only baseline and treatment information and no genetic data, which is a limitation that exists in this study^31–33^.

In AJCC staging, T stage represents the size and extent of the tumor, N stage represents lymph node metastasis, and M stage represents distant spread, with more stages representing more seriousness and worse prognosis. In this study, it was concluded that in N staging, breast cancer patients with N1 stage were more likely to develop secondary thyroid cancer compared to other stages, which may be related to the survival period, and we believe that only if the survival period is long enough, there is a possibility of developing a second primary tumor, while patients with N1 stage had lymph node metastasis, but it was not as serious as N2, N3, and N4 stage, so they were more likely to have a longer survival period; while patients with N0 stage had not No lymph node metastasis has occurred, and from the perspective of disease development, the possibility of secondary cancer is less than that of stage N1.In M stage, M0 stage represents no distant metastasis, and M1 stage represents distant metastasis, and we generally know that tumors with metastases are more likely to cause other cancers.

To summarize, in this study, univariate and multivariate cox analyses were used to screen the influencing factors of secondary thyroid cancer in breast cancer patients, respectively, and a column-line diagram was successfully established, and the calibration curves of the training and validation sets were well fitted, and the AUC and c-index reached significance. It is suggested that the column-line diagram has good predictive ability for the risk of secondary thyroid cancer in breast cancer patients 3 and 5 years after the onset of the disease. However, since the data were obtained from the United States, more studies are needed to verify whether the results obtained by applying this data can be applied to the Chinese population, and the results of this study can provide some references for clinicians^14^.

This study also has some limitations; first, this is a retrospective study, and selection bias due to incomplete data is inevitable. Although we used PSM to avoid selection bias, potential confounders cannot be excluded. Secondly, the Cox model fits well, but the AUC of the validation set is less than 0.7, which represents that the predictive ability of the model is still lacking, which may be related to the fact that breast cancer patients have a good prognosis and a long survival period, while only ten years of data have been observed in the present study, so in the future, we need more follow-up data to improve the model. Because the SEER database itself provides a limited amount of information and the database does not provide any information about genes, we were unable to study the genetic correlation of breast cancer secondary to thyroid cancer at the genetic level. The database has not been updated with data on tumor grading since 2017, so it is not possible to analyze the grading situation, which deserves further study in the future.

## Conclusions

In summary, our study suggests that breast cancer patients are more likely to develop thyroid cancer compared to the general population. Among them, age, ethnicity, marital status, primary tumor location, molecular typing, PR status, ER status, whether or not radiotherapy, whether or not chemotherapy, T stage, N stage, and M stage are the factors affecting the secondary thyroid cancer in breast cancer patients; age, ethnicity, whether or not radiotherapy was performed, primary location of the tumor, N stage, and M stage are the independent factors affecting the secondary thyroid cancer in patients with breast cancer patients: the younger the age, the whiter the ethnicity, the whites and the other ethnicities, undergoing radiotherapy, internal lower quadrant and other locations, N1 stage, and M1 stage are more likely to develop thyroid cancer in breast cancer patients.

## Data Availability

The datasets analyzed during the current study are available in the SEER*Stat software (version 8.4.2, download from https://seer.cancer.gov/data/options.html). A registration form needs to be completed before using and filtercriteria need to be added. The datasets are also available from the corresponding author on reasonable request.
